# Response times for reflexive saccades correlate with cognition in parkinson's disease, not disease severity or duration

**DOI:** 10.3389/fneur.2022.945201

**Published:** 2022-08-26

**Authors:** Yaqin Yu, Weihong Yan, Xin Xu, Kaili Zhang, Lihong Si, Xiaolei Liu, Jinyu Wang, Junling Song, Huanxin Sun, Xinyi Li

**Affiliations:** ^1^Department of Neurology, Third Hospital of Shanxi Medical University, Shanxi Bethune Hospital, Shanxi Academy of Medical Sciences, Tongji Shanxi Hospital, Taiyuan, China; ^2^Tongji Hospital, Tongji Medical College, Huazhong University of Science and Technology, Wuhan, China; ^3^Department of Neurosurgery, Chinese PLA General Hospital, Beijing, China

**Keywords:** Parkinson's disease, cognition, reflexive saccades, dementia, MMSE

## Abstract

**Objective:**

Dementia is a common and serious non-motor symptom in Parkinson's disease (PD). We aimed to investigate the reflexive saccade in PD patients and explore its potential role as a biomarker for cognitive decline.

**Methods:**

Using an infrared video-based eye tracker, we investigated reflexive saccades in 94 PD patients and 115 healthy controls (HCs). Saccadic parameters were compared between PD patients and HCs, and also among PD subgroups. The correlation of saccadic performance with disease duration, severity and cognition were further investigated.

**Results:**

Compared with healthy controls, PD patients had prolonged and hypometric reflexive saccades even in early disease stage. Univariate and multivariate analysis showed that there was significant inverse relation between prolonged latency and MMSE in PD patients (*P* < 0.05); tremor dominant PD patients were more likely to have decreased velocity than non-tremor-dominant PD patients (*P* < 0.05); saccadic accuracy was found to have no significant relation with disease duration, H&Y staging or MMSE.

**Conclusion:**

Reflexive saccadic performance was abnormal in PD and worsened with cognitive decline. The negative correlation between prolonged latency and MMSE scores may make the reflexive saccade a potential predictor for cognitive decline in Parkinson's disease.

## Introduction

Parkinson's disease dementia (PDD) and mild cognitive impairment (MCI) are the two most common cognitive syndromes in patients with Parkinson's disease (PD) (1, 2). According to the MDS Task Force, MCI, as the harbinger of dementia, is common in non-demented PD patients (mean prevalence 27%), but is often ignored by both family members and clinicians. Cumulatively 80% may develop dementia among PD patients with 20-years survival (3, 4). PDD patients have a faster motor progression and worse prognosis due to cognitive impairment. Early identification of cognitive decline may allow timely intervention to slow or prevent PDD (5). Therefore, biomarkers that can help identify cognitive decline as early as possible in PD patients are of great value in the clinic. Unfortunately, no biomarker has as yet been validated.

Recently, eye tracking technology has attracted much attention for its potentiality in identifying cognitive decline in neurological disorders. Saccades have been found to be associated with cognitive impairment in PD (6–9). Samuel Stuart found that pro-saccades may predict cognitive decline in several domains and be a useful non-invasive biomarker for long-term PD cognitive decline in early disease stage (10).

However, studies on saccades of PD patients have yielded paradoxical results. Voluntary saccades have been found to be markedly impaired in PD (11). Whereas, reflexive saccades are found to be relatively spared in some studies, while in others, are mildly abnormal (12, 13). Reflexive saccadic abnormality and its relevance to cognitive state in PD should be further clarified to see whether reflexive saccades can become a biomarker for the cognitive dysfunction in PD.

## Participants and methods

### Standard protocol approvals

This was a retrospective observational study which followed the Declaration of Helsinki Principles. The study protocol was approved by the Medical Ethics Committee of Shanxi Bethune Hospital in Shanxi, China. Informed consent was obtained from all participants involved in the study.

### Participants

94 PD patients and 115 healthy controls (HCs) were included in the study. PD patients were all recruited from the Neurology Clinic at Shanxi Bethune Hospital and were diagnosed either clinically established or clinically probable PD according to the 2015 MDS Clinical Diagnostic Criteria for Parkinson's Disease by the experienced movement disorders expert (14). There was no history of any known ophthalmological disorders in either group of participants and no known neurological abnormalities in the control group or any other neurological or neuropsychiatric disorders in the PD group.

Exclusion criteria for all participants were age < 30 years, being educated<6 years (to rule out the influence of the education level on cognitive evaluation), dementia (significant cognitive decline causing interference in occupational, domestic, or social functioning) (15), suspicion of a neurodegenerative disorder (other than PD), use of antipsychotic, anti-epileptic, antidepressant medication or sedative drugs within 72 h (these drugs may influence eye movement parameters) (16), a visual acuity < 0.4, blurry vision, diplopia, and visual field disturbances. An additional exclusion criterion for PD patients was severe tremor or dyskinesia, which might influence the accuracy of the test.

To prevent the impact of dopaminergic medication on eye movements, PD patients held their dopaminergic medication overnight before the test (17). All patients were assessed by a trained movement disorder specialist and recorded on video for assessment of MDS-UPDRS part -III score.

### Equipment

Eye movements were recorded using a head-mounted infrared video-based eye tracker at 174 Hz (InteracousticsVO425, DENMARK). All stimuli were presented on the display with a black background and data were collected by Interacoustics Video Nystagmography OtoAccessTMV.1.5 software. Participants were seated approximately 85 cm in front of the display on a chair with head keeping still by themselves or in the support of one family member in a quiet darkened room. The examiner and the family member (if needed) were outside the participants' field of view. The total duration of the eye movement tasks (including reflexive saccades, smooth pursuits and optokinetic nystagmus) was no more than 20 min. Before each test, the examiner instructed the participant verbally and performed a practice demonstration to ensure that the participant understood the oral instructions correctly and participants learned and tried once or twice before the final trial. Alertness was maintained by frequent verbal encouragement.

### Procedures

#### Clinical assessment

Participants' demographics including age, sex and level of education were recorded. PD duration was recorded in years since the first motor symptom appeared. Motor severity was assessed using the International Parkinson's and Movement Disorders Society Unified Parkinson's Disease Rating Scale (MDS-UPDRS) part-III, and Hoehn and Yahr Staging (H&Y) (18). Levodopa equivalent daily dose (LEDD) was calculated (19). Global cognition was measured using the Chinese version of the Mini mental State Examination (MMSE), which is the best-known and the most commonly used screening tool for the measurement of global cognitive impairment in clinics and research. The common threshold (scores ≤ 26) was used to divide PD patients into PD with normal cognition (PD-NC) subgroup and PD with impaired cognition (PD-IC) subgroup (20).

PD patients were also classified into tremor-dominant (TD), postural instability/gait difficulty (PIGD) and mixed subtypes clinically according to the ratio of mean tremor score to the mean postural instability/gait difficulty score (ratio ≥ 1.15 for TD,≤ 0.90 for PIGD, 0.90–1.15 for mixed) (21). PIGD and mixed subtypes were non-tremor-dominant (NTD) subtype.

#### Eye tracking procedure

A calibration sequence was performed at the beginning of the test by having the participant to look to five dots presented on the screen in a random sequence. Calibration was repeated during the tasks when the eye tracker slipped off the head or when participants needed a break from the eye tracker. In the reflexive saccadic task, participants were instructed to continuously fixate on a “jumping target” as accurately and quickly as possible for 30 s to 45 s. The target started in a central position, then disappeared and immediately reappeared at a random location in a horizontal plane with an interval of 200 ms and a variable distance from the previous location (2–30°), resulting in a total number of 15–20 trials.

### Outcome measures and statistical analysis

Primary outcome measures were mean gain (defined as primary saccade amplitude divided by target amplitude), mean peak velocity and mean latency or response time (defined as the time interval between target appearance and the start of a saccade). Mean latency ≥ 300 ms is defined as prolonged latency and mean velocity ≤ 520°/s is deemed as decreased velocity (The cutoffs are calculated from data collected from more than 600 healthy people aged over 30 years old examined by our equipment). Inaccurate saccades included hypometria (gain < 0.8) and hypermetria (gain > 1.1) (22). Data were presented as mean ± standard deviation (SD) or median (inter-quartile range) for continuous variables, and as frequency or percentage for categorical variables. In *post-hoc* analysis, PD patients were categorized by disease severity (H&Y staging), cognition and clinical subtypes respectively. Statistical analysis was conducted using the Statistical Package for Social Sciences (SPSS) software (version 22.0) and the statistical software packages R (http://www.R-project.org, The R Foundation) and Free Statistics software versions 1.3. Data were examined for normality of distribution with Kolmogorov-Smirnov's test. Student's *t*—test, Mann-Whitney test or one-way ANOVA was used to compare continuous variables. Differences between categorical variables were calculated using Pearson's chi-square test. Univariate logistic regression was used to estimate the odds ratios (ORs) and 95% CI for the abnormal saccades (including prolonged latency, decreased velocity and inaccuracy). Multivariate logistic regression was used to analyze all the related factors. A *p* value < 0.05 was considered to be statistically significant.

## Results

### General characteristics

PD patients and healthy controls were well–balanced in age and sex (*p* > 0.05). The median PD duration was 4.0(2.0, 7.8) years with varying levels of PD severity as measured by H&Y staging (stage I, 16%; stage II, 57%; stage III, 23%; stage IV, 3%; stage V 0%). The average MDS-UPDRS part-III score of PD patients in off-medication state was 39(29.0, 51.8). 42 PD patients had cognitive decline and 52 had normal cognition. PD patients had significant lower MMSE score than healthy controls (*p* < 0.001) (Table 1).

**Table 1:** Characteristics and reflexive saccadic parameters of participants.

	**HC**	**PD**	***p*** **-value**
	**(*n* = 115)**	**(*n* = 94)**	
Male (%)	48.7	59.6	0.153
Age (y)^*^	67.3 ± 9.4	65.2 ± 9.3	0.111
MMSE^**^	29 (29.0, 30.0)	27 (25.0, 28.0)	<0.001
LEDD^**^	NA	337.5 (0.0, 543.8)	
MDS-UPDRSIII^**^	NA	39.0 (29.0, 51.8)	
Duration (y) ^**^	NA	4.0 (2.0, 7.8)	
H&Y I (%)	NA	15.9	
H&Y II (%)	NA	57.5	
H&Y III (%)	NA	23.4	
H&Y IV (%)	NA	3.1	
Mean gain^**^	0.96 (0.89, 1.00)	0.90 (0.84, 0.96)	<0.001
Mean latency (ms) ^**^	304.5 (285.2,323.2)	322.8 (286.5,347.1)	0.005
Mean velocity (°/s) ^*^	612.1 ± 72.4	585.7 ± 100.0	0.055

* represented as mean ± standard deviation;

** represented as median (IQR); PD, Parkinson's disease, HC, healthy controls; MMSE, minimal mental state examination; LEDD, levodopa equivalent daily dose; H&Y, Hoehn and Yahr; MDS-UPDRS, Movement Disorder Society unified Parkinson's disease rating scale (version 3.0); NA, not applicable. P < 0.05 was considered statistically significant.

### Visually guided saccades

#### Comparison between PD group and HC group

**Figure 1:**
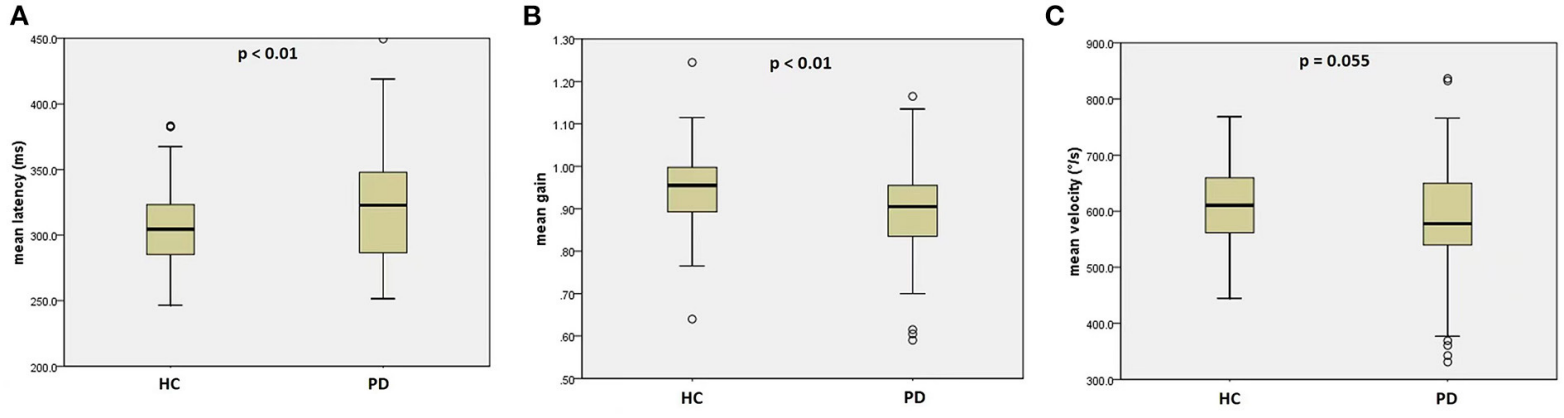
Reflexive saccades in the PD group and the HC group. **(A)** mean latency of reflexive saccades; **(B)** mean gain of reflexive saccades; **(C)** mean velocity of reflexive saccades. PD, Parkinson's disease, HC, healthy control.

Compared to healthy controls, PD patients had significantly prolonged mean latency (HC: 304.5 ms, PD:326.5 ms, *p* < 0.01), reduced mean gain (HC: 0.96, PD: 0.90, *p* <0.01), and a non-significant trend toward lower mean peak velocity (HC: 612.1 °/s, PD: 585.7°/s, *p* = 0.055), shown by Figures 1A–C. *Post-hoc* evaluation of reflexive saccadic accuracy showed that in PD patients, 24.3% of saccades were inaccurate, of which 80.6 % were hypometric. In HCs, only 2.7% of saccades were inaccurate: 67% hypometric and 31% hypermetric (χ^2^ = 20.2, *p* < 0.001).

#### Comparison between PD cognitive subgroups

**Figure 2:**
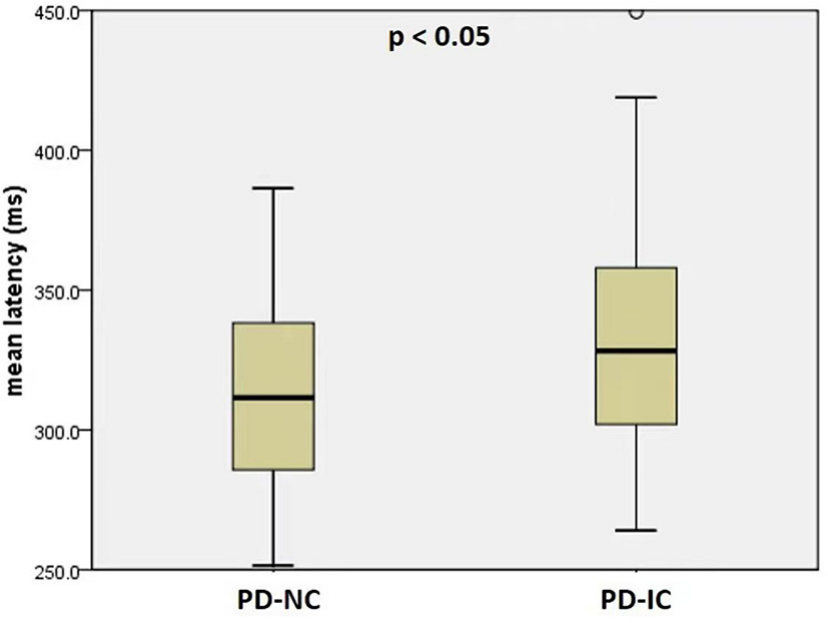
Mean latency of reflexive saccades in PD patients with normal cognition (PD-NC) and PD patients with impaired cognition (PD-IC).

PD patients with impaired cognition were much older in age (*p* = 0.026) and at higher H&Y stages (*p* = 0.023), and had higher MDS-UPDRS part-III score (*p* = 0.002) and lower MMSE score than PD patients with normal cognition (*p* < 0.001) (Table 2). There was no significant difference for mean disease duration or LEDD between the two PD cognitive subgroups (*p* = 0.292 and *p* = 0.341, resp.). PD-IC subgroup had significantly longer mean latency than PD-NC subgroup (PD-IC 332 ms, PD-NC: 312 ms, *p* < 0.05) (Figure 2). Mean gain and mean velocity did not differ significantly between the two subgroups (*p* = 0.9 and *p* = 0.4, resp.).

**Table 2:** Characteristics and reflexive saccadic parameters of PD cognitive subgroups.

	**Normal**	**Impaired**	***p*** **-value**
	**cognition**	**cognition**	
	***n*** **= 52**	***n*** **= 42**	
Male (%)	63.5	54.8	0.52
Age(y) ^*^	63.3 ± 9.3	67.6 ± 8.7	0.026
Duration (y) ^**^	4.0 (1.5, 6.2)	3.5 (2.0, 8.0)	0.292
LEDD^**^	337.5 (0.0, 500.0)	345.0 (150.0, 656.2)	0.341
MDS-UPDRSIII^*^	35.3 ± 15.5	46.0 ± 16.9	0.002
MMSE^**^	28.0 (27.0, 29.0)	25.0 (22.0, 25.0)	<0.001
Mean gain^*^	0.89 ± 0.10	0.89 ± 0.11	0.953
Mean velocity (°/s) ^**^	594.8 (545.2, 658.9)	564.2 (533.4, 639.5)	0.44
Mean latency (ms) ^*^	312.2 ± 35.6	332.3 ± 42.9	0.015

* represented as mean ± standard deviations,

** represented as median (IQR). PD, Parkinson's disease; MMSE, minimal mental state examination; LEDD, levodopa equivalent daily dose; H&Y, Hoehn and Yahr; MDS-UPDRS, Movement Disorder Society unified Parkinson's disease rating scale (version 3.0). P < 0.05 was considered statistically significant.

#### Comparison between PD clinical subgroups

Thirty-four PD patients were TD subtype and 60 were NTD subtype (including 51 PIGD and nine mixed subtype). TD and NTD patients were balanced in age, sex, duration, MDS-UPDRS part-III score and MMSE score (*p* > 0.05 for all). Higher LEDD and more patients at H&Y III and IV stages were found in non-tremor patients (*P* = 0.014 and *P* = 0.027, resp.). There was no significant difference for gain or latency between the two subgroups (*p* > 0.05 for both). NTD patients had higher velocity than TD patients (*p* = 0.025) (Table 3).

**Table 3:** Characteristics and reflexive saccadic parameters of PD clinical subtypes.

	**TD**	**NTD**	***p*** **-value**
	***n*** **= 34**	***n*** **= 60**	
Male (%)	67.6	55	0.326
Age (y) ^*^	65.9 ± 8.7	64.8 ± 9.6	0.59
Duration (y) ^**^	3.0 (2.0, 6.0)	5.0 (2.0, 8.0)	0.372
LEDD^**^	150.0 (0.0, 446.9)	375.0 (200.0, 681.2)	0.014
MDS-UPDRSIII^*^	39.3 ± 15.6	40.5 ± 17.7	0.738
MMSE^**^	27.0 (25.0, 28.0)	26.5 (25.0, 28.2)	0.623
H&Y (%)			0.027
I	20.6	13.3	
II	70.6	50	
III	8.8	31.7	
IV	0	5	
Mean gain^*^	0.9 ± 0.1	0.9 ± 0.1	0.485
Mean velocity (°/s) ^**^	553.0 (515.6, 617.4)	591.5 (550.0, 661.5)	0.025
Mean latency (ms) ^*^	317.9 ± 32.3	323.1 ± 44.1	0.556

* represented as mean ± standard deviations,

** represented as median (IQR). PD, Parkinson's disease; TD, tremor-dominant subtype; NTD, non-tremor-dominant subtype; MMSE, minimal mental state examination; LEDD, levodopa equivalent daily dose; H&Y, Hoehn and Yahr; MDS-UPDRS, Movement Disorder Society unified Parkinson's disease rating scale (version 3.0). P < 0.05 was considered statistically significant.

#### Comparison among PD patients at different H&Y stages

Significant differences were found in age, disease duration, MDS-UPDRS part-III score and MMSE score among H&Y stages I-IV (*p* = 0.014, *p* < 0.001, *p* < 0.001, and *p* = 0.003, resp.). However, there was no significant difference in mean gain, mean velocity or mean latency of reflexive saccades among different H&Y stages.

#### Correlation between latency and MMSE in PD patients

Sixty-one PD patients had prolonged latency and 33 had normal latency. There was significant difference in MMSE between PD patients with prolonged latency and PD patients with normal latency (*p* < 0.001). However, for age, sex, disease duration, H&Y staging or MDS-UPDRS part-III score, no significant differences were found between them (Table 4). In univariate logistic analysis there was significant inverse relation between prolonged latency and MMSE (Table 5). PD patients with higher MMSE scores were less likely to show prolonged reflexive saccadic latency (OR = 0.71; 95% CI 0.57–0.88; *p* = 0.002). Age (*p* = 0.142), sex (*p* = 0.243), MDS-UPDRS part-III scores (*p* = 0.994), disease duration (*p* = 0.497), and H&Y staging (*p* = 0.221 for stage II, *p* = 0.729 for stage III and *p* = 0.674 for stage IV) were not found to have significant relations with prolonged latency in PD patients. In multivariate logistic analysis, similar results were obtained (Table 5).

**Table 4:** Characteristics of PD patients with normal latency and prolonged latency.

	**PD with normal**	**PD with prolonged**	***p*** **-value**
	**latency**	**latency**	
	***n*** **= 33**	***n*** **= 61**	
Male (%)	51.5	63.9	0.342
Age (y) ^*^	63.3 ± 9.0	66.3 ± 9.3	0.14
Duration (y) ^**^	5.0 (3.0, 7.0)	3.0 (1.5, 8.0)	0.247
LEDD^**^	337.5 (0.0, 500.0)	345.0 (150.0, 656.2)	0.341
MDS-UPDRSIII^*^	40.1 ± 17.9	40.1 ± 16.5	0.995
MMSE^**^	28.0 (26.0, 29.0)	26.0 (24.0, 27.0)	<0.001
H&Y (%)			0.528
I	21.2	13.1	
II	48.5	62.3	
III	27.3	21.3	
IV	3	3.3	

* represented as mean ± standard deviations,

** represented as median (IQR). PD, Parkinson's disease; MMSE, minimal mental state examination; LEDD, levodopa equivalent daily dose; H&Y, Hoehn and Yahr; MDS-UPDRS, Movement Disorder Society unified Parkinson's disease rating scale (version 3.0). P < 0.05 was considered statistically significant.

**Table 5:** Univariate and multivariate analysis for the association between latency and possible factors.

	**Univariate results**		**Multivariate results**			
	**OR (95%CI)**	***p*** **-value**	**Adjust I**	***p*** **-value**	**Adjust II**	***p*** **-value**
MMSE	0.71 (0.57 ~ 0.88)	0.002	0.71 (0.57 ~ 0.89)	0.002	0.62 (0.48 ~ 0.82)	0.001
Age	1.04 (0.99 ~ 1.09)	0.142	1.01 (0.96 ~ 1.07)	0.64	1.02 (0.97 ~ 1.08)	0.405
Sex	1.67 (0.71 ~ 3.94)	0.243	1.91 (0.75 ~ 4.89)	0.175	2.09 (0.75 ~ 5.79)	0.156
Duration	0.96 (0.86 ~ 1.08)	0.497			0.93 (0.77 ~ 1.12)	0.446
MDS-UPDRSIII	1 (0.98 ~ 1.03)	0.994			0.96 (0.91 ~ 1.01)	0.138
H&Y						
I	Reference				Reference	
II	2.08 (0.64 ~ 6.7)	0.221			3 (0.47 ~ 18.96)	0.244
III	1.26 (0.34 ~ 4.75)	0.729			2.35 (0.17 ~ 32.59)	0.524
IV	1.75 (0.13 ~ 23.7)	0.674			1.65 (0.02 ~ 128.84)	0.821

data presented are ORs and 95% CIs. Adjust I model adjusts for age and sex; adjust II model adjusts for adjust I + disease duration, MDS-UPDRS-III, H&Y. MMSE, minimal mental state examination; H&Y, Hoehn and Yahr; MDS-UPDRS, Movement Disorder Society unified Parkinson's disease rating scale (version 3.0). P < 0.05 was considered statistically significant.

#### Correlation between velocity and PD clinical subtypes

Fifteen PD patients had decreased reflexive saccadic velocity and 79 had normal velocity. There were no significant differences in age, sex, disease duration, MMSE, H&Y staging, MDS-UPDRS part-III score or clinical subtypes between PD patients with normal velocity and PD patients with decreased velocity. In univariate logistic analysis, NTD patients were less likely to show decreased velocity (OR = 0.31; 95% CI 0.1 ~ 0.96; *p* = 0.043). Age (*p* = 0.676), sex (*p* = 0.592), MDS-UPDRS part-III scores (*p* = 0.319), disease duration (*p* = 0.841), LEDD (*p* = 0.903), and H&Y staging (*p* = 0.29 for stage II, *p* = 0.511 for stage III, and *p* = 0.225 for stage IV) were not found to have significant relations with reflexive saccadic velocity in PD patients. In multivariate logistic analysis, after adjusting sex, age, disease duration, LEDD, H&Y staging, and MDS-UPDRS part-III scores, results analogous to univariate analysis were gained (Table 6).

**Table 6:** Univariate and multivariate analysis for the association between velocity and possible factors.

	**Univariate results**		**Multivariate results**			
	**OR (95%CI)**	***p*** **-value**	**Adjust I**	***p*** **-value**	**Adjust II**	***p*** **-value**
**Clinical subtypes**						
TD	Reference		Reference		Reference	
NTD	0.31 (0.1 ~ 0.96)	0.043	0.29 (0.09 ~ 0.92)	0.036	0.18 (0.04 ~ 0.74)	0.018
Age	1.01 (0.95 ~ 1.08)	0.676	1.01 (0.95 ~ 1.08)	0.731	0.99 (0.92 ~ 1.06)	0.745
Sex	0.74 (0.24 ~ 2.24)	0.592	0.61 (0.19 ~ 1.94)	0.401	0.47 (0.13 ~ 1.65)	0.238
Duration	1.02 (0.87 ~ 1.18)	0.841			0.99 (0.81 ~ 1.22)	0.946
MDS-UPDRSIII	1.02 (0.98 ~ 1.05)	0.319			0.99 (0.94 ~ 1.05)	0.76
LEDD	1 (1 ~ 1)	0.903			1 (1 ~ 1)	0.625
**H&Y**						
I	Reference				Reference	
II	3.18 (0.37 ~ 27.09)	0.29			5.1 (0.36 ~ 71.97)	0.228
III	2.21 (0.21 ~ 23.56)	0.511			7.84 (0.23 ~ 271.39)	0.255
IV	7 (0.3 ~ 162.21)	0.225			62.47 (0.4 ~ 9,774.42)	0.109

data presented are ORs and 95% CIs. Adjust I model adjusts for age and sex; adjust II model adjusts for adjust I + LEDD, disease duration, MDS-UPDRSIII and H&Y.TD, tremor-dominant subtype; NTD, none tremor-dominant subtype; MMSE, minimal mental state examination; LEDD, levodopa equivalent daily dose; H&Y, Hoehn and Yahr; MDS-UPDRS, Movement Disorder Society unified Parkinson's disease rating scale (version 3.0). P < 0.05 was considered statistically significant.

#### Correlation between accuracy and related factors

Nineteen PD patients had inaccurate saccades (17 hypometric and 2 hypermetric) and 75 had accurate saccades. Age and sex were well–matched between PD patients with inaccurate saccades and PD patients with accurate saccades and significant differences in disease duration (*p* = 0.003), MMSE (*p* = 0.041), H&Y staging (*p* = 0.001), MDS-UPDRS part-III score (*p* = 0.008), and LEDD (*p* < 0.001) were found between them. In univariate logistic analysis, disease duration (OR = 1.25; 95%CI 1.09~1.44; *p* = 0.002), MMSE (OR = 0.8; 95%CI 0.66 ~ 0.96; *p* = 0.017) and MDS-UPDRS part-III score (OR = 1.04; 95%CI 1.01~1.08; *p* = 0.012) were found to be significantly related to saccadic accuracy, while in multivariate analysis, saccadic accuracy was not found to have significant relations with them.

## Discussion

To the best of our knowledge, this is the first study to investigate reflexive saccades in a large number of PD patients in off-medication state and the reflexive saccade–to-cognition relationship in PD.

Main findings were: first, PD patients presented prolonged and hypometric reflexive saccades; second, the likelihood of prolonged latency was inversely correlated with MMSE score in PD and low MMSE score was an independent risk factor of prolonged latency; thirdly, TD patients were more likely to show decreased velocity than non-tremor dominant PD patients.

Cognitive deficits are common in PD, even in prodromal or early stage of the disease (1). Therefore, biomarkers that can identify early cognitive decline are desperately needed. Saccadic eye movements have been one of the attractive candidates for use in early cognitive decline recognition (6, 7, 10, 23). Reflexive saccadic tasks were taken in our study, because of the controversial results and better cooperation by PD patients. We found that reflexive saccades were not only abnormal in PD patients even in early disease stage, but also significantly correlated with cognition.

### Latency of reflexive saccades

Individuals with PD show marked impairments in the initiation of voluntary saccadic eye movements (11, 24). However, investigations of reflexive saccades in PD have, on the other hand, presented inconsistent results with prolonged, shortened or indistinguishable latency. Possible reasons might be the small number of participants and considerable heterogeneity among these studies, in which disease severity, medication, target eccentricities, eye-tracking and display equipment were all varied (13). PD patients in our study had significantly prolonged reflexive saccadic latency than healthy controls and among PD patients, prolonged latency was inversely related to MMSE.

Reflexive saccades are initiated directly from the frontal and parietal cortex onto saccade-related neurons in the intermediate layer of the superior colliculus (SC), which then project to the brainstem saccade generators, bypassing the basal ganglia (24). The abnormalities of reflexive saccades in PD reflect the direct cortical-SC circuit dysfunction even in early stage of PD. Although the basal ganglia are not involved in the generation of reflexive saccades directly, SC is excessively inhibited as a result of dopamine depletion in the basal ganglia, which is caused by the degeneration of substantia nigra pars compacta (SNc) in PD. As the final common pathway for both reflexive and voluntary saccades, the SC is involved in initiation and inhibition of saccades and activity of the SC is negatively related to saccade latency. The stronger the activity, the faster eyes move to the target, thus the shorter the latency will be (12, 24, 25). Excessive inhibition of the SC may have resulted in the abnormal reflexive saccades in the PD group in our study. PD is the aggregation of a-synuclein inclusions in the neurons, which has been proved to spread from the vagus nerve to the medulla oblongata and pontine tegmentum before reaching the midbrain (26). Lewy bodies and degeneration of dopaminergic neurons in the SNc are the neuropathological hallmarks of PD. By the time that clinical symptoms become evident, SNc dopaminergic neurons are at least 50–70% lost (27). Therefore, the early stage in clinic is rather an advanced stage in pathology, which could explain the abnormal reflexive saccades even in early disease stage clinically. According to Braak and his coworkers, PD is a multisystem disorder and with disease progression, the classical pathology may continue spreading from the midbrain to the thalamus and cortex, resulting in psychiatric problems and dementia (26). Thus the cortical -SC circuit dysfunction will be further aggravated due to the cortical involvement, which could explain what we have found that PD patients with cognitive dysfunction are more likely to show prolonged latency.

### Velocity of reflexive saccades

Consistent with previous studies, no significant difference in the reflexive saccadic velocity was found between PD patients and healthy controls (22, 28). However, between PD clinical subtypes, reflexive saccadic velocity was found to differ significantly, with TD patients having a significantly lower velocity than NTD patients, which has not been reported before. There was no significant difference in basic clinical characteristics between the two subgroups, except the lower LEDD and less patients at H&Y III and IV stages in TD patients. However, analysis showed that there was no significant difference for reflexive saccadic performance among different H&Y stages.

Parkinson's disease is a clinically heterogeneous disease with bradykinesia, rigidity, and rest tremor being the cardinal triad and presenting in a various proportion (27). It has been proved by increasing clinical and experimental evidence that TD and PIGD subtypes have different clinical courses and pathologies, and are not generated by identical neuronal mechanisms. In clinic, tremor-dominant patients show less cognitive decline and depressive symptoms, less responsiveness to dopaminergic medication, a slower progress and a relatively better prognosis (29). Pathologically, the PIGD subtype shows more severe cell loss in the ventrolateral SNc that projects to the dorsal putamen and the TD subtype presents a more severe neuronal loss in medial SNc, which projects to the caudate nucleus and anterior putamen. Moreover, the retrorubral area with projections to the dorsolateral striatum and ventromedial thalamus is more severely affected in the TD subtype. In pathogenesis, the dysfunction of striatal-thalamo-cortical (STC) circuit is involved in bradykinesia and rigidity, while rest tremor is due to the dysfunction of cerebello-thalamo-cortical (CTC) circuitry (30, 31). Thus TD group having a much lower velocity may be due to the more severe and widely affected pathology and dual involvement of STC and CTC circuits. However, detailed mechanism for this phenomenon awaits further research to elucidate.

### Gain of reflexive saccades

Saccadic hypometria is one of the most consistent findings in PD, which presents a series of discrete short saccades before reaching the target, referred to as a multiple step pattern (24, 32). In our study, PD patients showed mild hypometric reflexive saccades, which were irrelevant to disease severity or duration. Cerebellum plays an important function in accuracy and calibration of the saccade, which explains the fact that MSA patients show moderate to severe hypometria, whereas PD patients show only mild hypometria. Hypometria in PD may be due to the irregular pulse in omnipause neurons of the brainstem or cortical dysfunction, whereas hypometria and hypermetria in MSA are mainly attributed to impairment of the cerebellum (33–35).

### Limitations

There are limitations of the present study. First, there were few patients at higher H&Y stages (three at stage IV and none at stage V) and this could lead to bias. Sample sizes can be expanded to comprise more patients at higher H&Y stages. However, it may be difficult to investigate advanced patients in off-medication state either because patients are too severe in off-medication state to cooperate or because patients cannot hold their medications overnight. Second, the reflexive saccadic amplitude in our study varied widely between 2 and 30°. Further studies can be carried out to analyze the influence of small and large saccades on PD patients separately. Thirdly, mild cognitive impairment in patients with Parkinson's disease is a heterogeneous entity that involves different types and extents of cognitive deficits. MMSE, though commonly used in clinic, lacks the executive domain. Other tests, like the Montreal Cognitive Assessment (MoCA), are more informative and sensitive in neurodegenerative disorders, should be used in future studies to elucidate the influence of the impairment of global cognition and its subdomains on the eye movements in PD patients. And it's worthwhile to conduct a prospective study on the saccadic performance and cognitive functions longitudinally in the course of PD to establish the potential function of reflexive saccades as a biomarker for cognitive decline in PD.

## Conclusion

Parkinson's disease patients presented reflexive saccadic abnormalities even in early disease stage. Prolonged latency of the reflexive saccades was in a negative correlation with MMSE scores, which were in line with cortical-SC circuit dysfunction in PD. The velocity difference in clinical subgroups further supported the heterogeneity and the pathogenesis variation between PD subtypes. Reflexive saccades may be a potential biomarker for early cognitive decline.

## Data availability statement

The raw data supporting the conclusions of this article will be made available by the authors, without undue reservation.

## Ethics statement

The studies involving human participants were reviewed and approved by the Medical Ethics Committee of Shanxi Bethune Hospital in Shanxi, China. The patients/participants provided their written informed consent to participate in this study.

## Author contributions

YY and XX: conceptualization. YY: data curation and drafting manuscript. YY, WY, XL, JW, JS, and HS: project administration. YY, LS, and KZ: statistical analysis. XL: funding acquisition, reviewing the manuscript and supervision. All authors have read and agreed to the published version of the manuscript. All authors contributed to the article and approved the submitted version.

## Funding

Research Project Supported by Shanxi Scholarship Council of China (No. HGKY2019096).

## Conflict of interest

The authors declare that the research was conducted in the absence of any commercial or financial relationships that could be construed as a potential conflict of interest.

## Publisher's note

All claims expressed in this article are solely those of the authors and do not necessarily represent those of their affiliated organizations, or those of the publisher, the editors and the reviewers. Any product that may be evaluated in this article, or claim that may be made by its manufacturer, is not guaranteed or endorsed by the publisher.
